# 

**Published:** 1918

**Authors:** 


### THE BRISTOL

### Medico-Chirurgical Journal.

A JOURNAL OF THE MEDICAL SCIENCES FOR THE
WEST OF ENGLAND AND SOUTH WALES.

*PUBLISHED UNDER THE AUSPICES OF*

*THE BRISTOL MEDICO-CHIRURGICAL SOCIETY.*

EDITOR:
P. WATSON-WILLIAMS, M.D.

WITH WHOM ARE ASSOCIATED

J. LACY FIRTH, M.S., M.D., JAMES SWAIN, C.B., C.B.E., M.S., M.D.,
CAREY COOMBS, M.D.

J. A. NIXON, C.M.G., M.D., ASSISTANT EDITOR.

EDITORIAL SECRETARY:
J. M. FORTESCUE-BRICKDALE, M.A., M.D.

"Scire est nescire, nisi id me

Scire alius sciret."

*VOL. XXXVI.*

BRISTOL: J. W. ARROWSMITH LTD.
LONDON: SIMPKIN, MARSHALL, HAMILTON, KENT & CO. LIMITED.
1919.

R31
.B8

J. W. Arrowsmith Ltd.

Publishers

Bristol

### CONTENTS OF VOLUME XXXVI.

| | Page |
|---|---|
| Some Developments in Abdominal Surgery. By F. LACE, F.R.C.S. .. | 1 |
| The Systematic Examination of the Abdomen. By R. G. P. LANSDOWN, M.B., B.S. Durh. .. .. .. .. .. | 33 |
| Postgraduate Study. By J. M. FORTESCUE-BRICKDALE, M.A., M.D., M.R.C.P. .. .. .. .. .. .. .. .. | 48 |
| The Operative Findings in Thirty Cases of Gunshot Injury of Nerves, with Remarks on Diagnosis, Localisation, and the Technique of Operation. By C. A. MORTON, F.R.C.S. .. .. .. | 55 |
| Contemporary Medicine from the Standpoint of Pathology. By I. WALKER HALL, M.D. .. .. .. .. .. | 105 |
| Disease in Mesopotamia. By Major F. P. MACKIE, M.D., M.Sc., F.R.C.S., F.R.C.P., I.M.S. .. .. .. .. .. .. | 118 |
| On the Treatment of Ununited Fractures, with Especial Reference to the Use of Bone Grafts. By ERNEST W. HEY GROVES, M.S., F.R.C.S. .. .. .. .. .. .. .. .. | 132 |
| Stammering as it Occurred in the War. By R. G. GORDON, M.D., B.Sc., M.R.C.P. Ed. .. .. .. .. .. .. | 143 |
| PROGRESS OF THE MEDICAL SCIENCES .. .. .. .. .. | 151 |
| REVIEWS OF BOOKS .. .. .. .. .. .. | 81, 162 |
| EDITORIAL NOTES .. .. .. .. .. .. .. .. | 18, 87 |
| THE LIBRARY OF THE BRISTOL MEDICO-CHIRURGICAL SOCIETY | 93, 171 |
| MEETINGS OF SOCIETIES .. .. .. .. .. .. .. | 100, 174 |
| OBITUARY .. .. .. .. .. .. .. | 23, 100, 177 |
| LOCAL MEDICAL NOTES .. .. .. .. .. .. .. | 103, 183 |

3m 26/VIII/22

### Bristol Medico-Chirurgical Society.

### President.

FREDERICK LACE, F.R.C.S. Eng., L.R.C.P. Lond.

### President-Elect.

R. G. P. LANSDOWN, M.B., B.S.Durh.

### Committee.

*Ex-Officio.*
- F. H. EDGEWORTH, M.A., M.D. Cantab., D.Sc. Lond. (Retiring President).
- P. WATSON-WILLIAMS, M.D. Lond. (Editor of *Journal*).
- C. K. RUDGE, M.R.C.S. (Hon. Librarian).
- J. M. FORTESCUE-BRICKDALE, M.A., M.D., B.Ch.Oxon. (Editorial Secretary).

*Elected.*

GEORGE PARKER, M A., M.D. Cantab. } Ex-Presidents.

R. SHINGLETON SMITH, M.D., B.Sc. Lond. } Ex-Presidents.

J. WALKER HALL, M.D. Vict.

J. LACY FIRTH, M.D., M.S. Lond.

J. O. SYMES, M.D. Lond.

L. E. V. EVERY-CLAYTON, M.D., B.S. Lond.

W. A. SMITH, M.A., M.B.

THOS. CARWARDINE M.S.

### Acting Honorary Secretary.

A. L. FLEMMING, M.B., Ch.B. Bristol.

Medical Practitioners wishing to join the Society are requested to communicate with the Acting Hon. Secretary, Dr. A. L. FLEMMING, 48 Pembroke Road, Clifton. The Annual Subscription is 21/-.

---

*Extracts from Journal By-laws.*

4.—The *Journal* shall be delivered free to Subscribers and also to Members of the Society whose subscriptions are not in arrear.

6.—The Annual Subscription to the *Journal* for those who pay before the 1st of July shall be 5/-, post free, or 6/- if paid after that date.

---

Communications intended for insertion in the *Journal* should be sent to the Editor, Dr. WATSON-WILLIAMS, 2 Rodney Place, Clifton, Bristol.

Books for Review and Exchange Journals should be sent to the Assistant-Editor, *Bristol Medico-Chirurgical Journal*, Medical Library, The University, Bristol.

Communications referring to the delivery of the *Journal* should be sent to the Acting Editorial Secretary, Dr. W. A. SMITH, 70 Pembroke Road, Clifton, Bristol, to whom Subscriptions are to be paid *in advance*.

Cases for Binding Vols. I—XXXV are now ready, and may be obtained, price 1/4 each (postage 2*d*. extra), upon application to J. W. ARROWSMITH LTD., Quay Street, Bristol; or the *Journal* will be bound, including Case, for 2/6 per volume.

Owing to the war, no March or September issue of the 1916 *Journal* was published, so that Vol. XXXIV consisted of the two special parts numbered 130, 131, and only three parts were issued in 1917, so tha Vol. XXXV consists of numbers 132, 133 and 134.

### Bristol Medico-Chirurgical Society.

### PAST PRESIDENTS.

1874—75. FREDERICK BRITTAN, M.D., B.A. Dub.
1875—76. ROBERT WILLIAM COE.
1876—77. SAMUEL MARTYN, M.D. Ed.
1877—78. AUGUSTIN PRICHARD, M.D. Berlin.
1878—79. HENRY EDWARD FRIPP, M.D. Würzburg.
1879—80. WILLIAM MICHELL CLARKE.
1880—81. JOSEPH GRIFFITHS SWAYNE, M.D. Lond.
1881—82. EDWARD LONG FOX, M.D. Oxon.
1882—83. JAMES GEORGE DAVEY, M.D. St. And.
1883—84. WILLIAM JOHNSTONE FYFFE, M.D., B.A. Dub.
1884—85. GEORGE FORSTER BURDER, M.D. Aberd.
1885—86. WILLIAM HENRY SPENCER, M.D., M.A. Cantab.
1886—87. FRANCIS POOLE LANSDOWN.
1887—88. LEMUEL MATTHEWS GRIFFITHS.
1888—89. ROBERT SHINGLETON SMITH, M.D., B.Sc. Lond.
1889—90. NELSON CONGREVE DOBSON, Ch.M. Bristol.
1890—91. SAMUEL HENRY SWAYNE.
1891—92. FRANCIS RICHARDSON CROSS, M.B.Lond., LL.D.Bristol.
1892—93. EDWARD MARKHAM SKERRITT, M.D., B.S., B.A. Lond.
1893—94. JAMES GREIG SMITH, M.B., C.M., M.A. Aberd., F.R.S.E.
1894—95. ALFRED JAMES HARRISON, M.B. Lond.
1895—96. ARTHUR WILLIAM PRICHARD.
1896—97. ALFRED EDWARD AUST LAWRENCE, M.D., C.M. Aberd.
1897—98. JOHN EDWARD SHAW, M.B., C.M. Ed.
1898—99. ROBERT ROXBURGH, M.B. Ed.
1899—00. WILLIAM HENRY HARSANT.
1900—01. DAVID SAMUEL DAVIES, M.D. Lond.
1901—02. BARCLAY JOSIAH BARON, M.B., C.M. Ed.
1902—03. GEORGE MUNRO SMITH, M.D. Bristol.
1903—04. JAMES PAUL BUSH, C.M.G., Ch.M. Bristol.
1904—05. REGINALD EAGER, M.D. Lond.
1905—06. JOHN DACRE.
1906—07. JAMES TAYLOR.
1907—08. HENRY WALDO, M.D., C.M. Aberd.
1908—09. JOHN MICHELL CLARKE, M.D., M.A. Cantab.
1909—10. JAMES SWAIN, M.D., M.S. Lond.
1910—11. BERTRAM MITFORD HERON ROGERS, M.D., B.Ch., B.A. Oxon.
1911—12. CHARLES ALEXANDER MORTON.
1912—13. WALTER CARLESS SWAYNE, M.D., B.S. Lond. and Bristol.
1913—14. PATRICK WATSON-WILLIAMS, M.D. Lond.
1914—15. WILLIAM HARRY CHRISTOPHER NEWNHAM, M.A., M.B. Cantab.
1915—16. GEORGE PARKER, M.A., M.D. Cantab.
1916—17. FRANCIS HENRY EDGEWORTH, M.A., M.D. Cantab., D.Sc. Lond.

1918.

---

### LIST OF SUBSCRIBERS

TO THE

### Bristol Medico-Chirurgical Journal.

---

### HONORARY MEMBERS OF THE SOCIETY.

| | |
|---|---|
| Owen, Sir Isambard, D.C.L., M.D. | Hereford House, Clifton. |
| Dobson, N. C., Ch.M., F.R.C.S. ... | 16 College Road, Clifton. |
| Griffiths, L. M., M.R.C.S. ... ... | 11 Pembroke Road, Clifton. |
| Smith, R. Shingleton, M.D., B.Sc., F.R.C.P. ... ... ... ... ... ... | Deepholm, Clifton Park, Clifton. |

---

### SUBSCRIBERS.

*Those marked * are Members of the Society.*

| | |
|---|---|
| Ackland, J. McKno ... ... ... ... ... ... | 1 Barnfield Crescent, Exeter. |
| *Ackland, W. R., M.D.S. Bristol ... ... ... | 5 Rodney Place, Clifton. |
| Adye, W. J. A. ... ... ... .. ... ... ... | St. Margarets, Bradford-on Avon, Wilts. |
| Aldous, George F. ... ... ... ... ... ... | Charlton House, Mannamead, Plymouth. |
| *Alexander, D.A., M.B., Ch.B. Vict.... ... ... | 30 Berkeley Square, Clifton. |
| Alford, H. J., M.D. Lond.... ... ... ... ... | 19 Park Street, Taunton. |
| *Almond, G. H. H., M.A., M.B., B.Ch. Oxon. | 6 Brock St., Bath. |
| *Askins, R.A., M.D., B.Ch., B.A.O. Dubl. ... | Guildhall, Bristol. |
| *Aubrey, T., M.B. Lond. ... ... ... ... ... | Bitton, near Bristol. |
| Aveline, H. T. S., M.D. Durh. ... ... ... ... | Somerset Asylum, Taunton. |
| *Baker, Lily A., M.D., B.Ch., B.A.O., R.U.I.... | 8 Richmond Hill, Clifton. |
| *Ballance, H. Stanley M.D. Lond. ... ... | 1 Royal Terrace, Weston-super-Mare. |
| Barber, M. C., M.B., Ch.B. Bristol ... ... | Ingledene, High Street, Staple Hill Bristol. |
| *Barker, G. H., M.D., B.Sc. Lond. ... ... ... | 124 Redland Road, Bristol |
| *Baron, Sir Barclay J., M.B. C.M. Ed. ... | 16 Whiteladies Road, Clifton. |

### LIST OF SUBSCRIBERS.

| | |
|---|---|
| *Benson, J. R. ... ... ... ... ... ... ... ... | Fiddington House, Market Lavington, Wilts. |
| *Bergin, F. G. ... ... ... ... ... ... ... ... | 31 Oakfield Road, Clifton. |
| Bernard, C. ... ... ... ... ... ... ... .. ... | 704 Fishponds Road, Fishponds, Bristol. |
| Berry, F. C., M.D., B.Ch., B.A. Dub. ... ... | Locarno, Burnham, Somerset. |
| Berry, James, M.B., B.S. Lond. .. ... ... | 21 Wimpole Street, London, W.1. |
| Biamonti, Signor Sebastiano ... ... ... ... | 27 Via Balbi, Genoa, Italy. |
| *Blacker, A. E.... ... ... ... ... ... ... ... | 20 Victoria Square, Clifton. |
| Blackett, J. F., M.D., B.S. Lond. ... ... ... | 10 South Stoke Road, Combe Down, Bath. |
| *Blagg, Arthur F., M.D. Durh.... ... ... ... | 28 Caledonia Place, Clifton. |
| Bodman, J. Hervey, M.D. Lond. ... ... ... | 9 Whiteladies Road, Clifton. |
| *Bowker, G. E., M.D. Edin.... ... ... ... ... | 11 North Parade, Bath. |
| *Brasher, C. W. J., M.B., Ch.B. Bristol ... ... | 17 Oakfield Road, Clifton. |
| *Bristowe, H. C., M.D. Lond. ... ... ... ... | Wrington, Somerset. |
| *Brown, William, M.D., C.M. Glasg. ... ... | Park View, Fishponds, Bristol. |
| *Burdon-Cooper, J., M.D., B.Sc. Durh. ... ... | 12 The Circus, Bath. |
| Burroughs, E. F. H. ... ... ... ... ... ... | 27 Burlington Road, Redland, Bristol. |

| | |
|---|---|
| *Cadman, E. S. R., M.D., C.M. Edin. ... ... | Gordon House, Westham, Pevensey, Sussex. |
| *Cairns, F. J. ... ... ... ... ... ... ... ... ... | Devon House, Whitehall Road, Bristol. |
| *Carling, A., M.A., M.B., B.C. Cantab. ... ... | 12 Great George Street, Bristol. |
| *Carter, T. M., M.D. Durh. ... ... ... ... | Lieut.-Col., R.A.M.C. |
| *Carwardine, Thomas, M.B., M.S. Lond. ... | 16 Victoria Square, Clifton. |
| *Cave, Edward J., M.D. Lond. ... ... ... ... | 20 Circus, Bath. |
| *Charles, J. R., M.A., M.D., B.C. Cantab. ... | 12 Victoria Square, Clifton. |
| *Chitty, H., M.S. Lond. ... ... ... ... ... | 46 Pembroke Road, Clifton. |
| *Christofferson, P. E., M.B., Ch.B. Bristol .. | 8 Lansdown Place, Clifton. |
| Chute, Henry M. ... ... ... ... ... ... ... ... | King William's Town, S. Africa. |
| *Clarke, C., M.B., B.S. Lond. ... ... ... ... | Brompton Hospital, London, S.W. |
| *Clarke, R. C., M.B., Ch.B. Bristol ... ... ... | Amherst House, Clifton Park, Clifton. |
| Clowes, E. F.... ... ... ... ... ... ... ... ... | |
| Connellan, Lieut. P. S., I.M.S. ... ... ... | c/o Grindlay & Co., 54 Parliament Street, London, S.W. |
| *Cooke, H. W. ... ... ... ... ... ... ... ... ... | Lindthorpe, Southville, Bristol. |
| *Coombs, Carey, M.D. Lond. ... ... ... ... | 3 Pembroke Road, Clifton. |
| *Cooper, William Russell ... ... ... ... | 13 Westfield Park, Redland, Bristol. |
| *Corfield, C. .. ... ... ... ... ... ... ... | 12 Pembroke Road, Clifton. |
| Cossham, W. R., M.D., C.M. Aberd.... ... ... | Wellesley House, Cirencester. |
| Cotton, William, M.D., C.M., M.A. Ed. ... | 231 Gloucester Road, Bristol. |
| Coulter, R. J., M.B. Dub. ... ... ... ... ... | Ferncliffe, Stow Park Crescent, Newport, Mon. |
| Cridland, A. B. ... ... ... ... ... ... ... ... | Salisbury House, Chapel Ash, Wolverhampton. |
| *Cross, F. R., M.B. Lond., LL.D. Bristol ... | Worcester House, Clifton. |
| *Crossman, F. W., M.B.... ... ... ... ... ... | White's Hill, Hambrook, near Bristol. |
| *Crouch, C. Percival, M.B. Lond. ... ... ... | Villa Rosa, Weston-super-Mare. |

### LIST OF SUBSCRIBERS.

| | |
|---|---|
| *Dacre, John ... ... ... ... ... ... ... ... | 14 Eaton Crescent, Clifton. |
| *Davies, D. S., M.D. Lond., D.P.H. Cantab. ... | 6 Lansdown Place, Clifton. |
| Dening, Edwin ... ... ... ... ... ... ... | Manor House, Stow-on-the-Wold, Glos. |
| Devine, H., M.D., Lond. ... ... ... ... ... | Corporation Mental Hospital, Portsmouth. |
| *Dixon, T. B. ... ... ... ... ... ... ... ... | 134 Coronation Road, Bristol. |
| *Dowling, E. A. G. ... ... ... ... ... ... | 5 Rodney Place, Clifton. |
| *Drapes, T. L., M.B., B.C., B.A. Cantab. ... | St. Maur, Beaufort Square, Chepstow. |
| *Duckett, A. H., M.B. ... ... ... ... ... ... | Hapsford House, South Stoke Road, Combe Down, Bath. |
| Dunbar, Eliza L. Walker, M.D. Zürich ... | 9 Oakfield Road, Clifton. |
| Dymoke, F. ... ... ... ... ... ... ... ... | 21 Cotham Road, Bristol. |
| *Edgeworth, F. H., M.D., B.C., M.A. Cantab., D.Sc. Lond. ... ... ... ... ... ... ... ... | 4 Richmond Park Road, Clifton. |
| Edwards, C. D., B.A., M.D. Cantab. ... ... | The Corderies, Chalford, Glos. |
| Elliot Bros. Ltd. ... ... ... ... ... ... | *Australian Pharmaceutical Notes & News*, O'Connell Street, Sydney, N.S.W. |
| *Elliott, Christopher, M.D., B.A. Dub. ... | 102 Pembroke Road, Clifton. |
| Elliott, F. Percy, M.B., C.M. Aberd. ... ... | 113 Grove Rd., Walthamstow, London, E.17. |
| *Eskell, B.C., M.B., Ch.B. Bristol ... ... ... | |
| *Every-Clayton, L. E. V., M.D. Lond ... ... | Emsworth House, Clevedon, Somerset. |
| *Faill, C. J. C. ... ... ... ... ... ... ... ... | 40 Princes Street, Bristol. |
| Farnfield, W. W. ... ... ... ... ... ... ... | Clive Vale, Gillingham, Dorset. |
| *Fells, A., M.B., C.M. Edin. ... ... ... ... | 10 Elton Road, Clifton. |
| *Firth, J. L., M.D., M.S. Lond. ... ... ... ... | 8 Victoria Square, Clifton. |
| *Flemming, A. L., M.B., Ch.B. Bristol ... ... | 48 Pembroke Road, Clifton. |
| *Flemming C. E. S. ... ... ... ... ... ... | Manvers House, Bradford-on-Avon. |
| *Fortescue-Brickdale, J. M., M.A., M.D., B.Ch. Oxon. ... ... ... ... ... ... ... ... | 52 Pembroke Road, Clifton. |
| Foss, E. V., M.D., Ch.B. Bristol ... ... ... | Clouds Hill House, St. George, Bristol. |
| *Fraser, Forbes ... ... ... ... ... ... ... | 5 The Circus, Bath. |
| Freeman, J., M.D. Brux. ... ... ... ... ... | Waterloo Villa, 105 Queen's Road, Clifton. |
| *Galpin, P. A., M.D., B.S. Durh. ... ... ... | 170 Browning Road, Manor Park, London, E.12. |
| Garland, E. B., M.B., C.M. Edin. ... ... ... | 114*a* Hampton Road, Redland, Bristol. |
| *Gerrish, D. S., M.R.C.S. Lond.... ... ... ... | 328 & 330 Church Road, St. George, Bristol. |
| *Gibbs, A. N. Godby ... ... ... ... ... ... | 52 Whiteladies Road, Clifton. |
| *Gratte, C. Brooke ... ... ... ... ... ... | 18 Gold Tops, Newport, Mon. |
| *Green, T. A., M.D., C.M. Edin. ... ... ... | 33*a* Whiteladies Road, Clifton. |
| Green-Armytage, Capt. V. B., I.M.S. ... ... | c/o T. Cook & Son, Calcutta. |
| *Greer, W. J. ... ... ... ... ... ... ... ... | 19 Gold Tops, Newport, Mon. |
| Griffiths, Cornelius A. ... ... ... ... ... | 35 Newport Road, Cardiff. |
| *Griffiths, J. S. ... ... ... ... ... ... ... | 20 Redland Park, Bristol. |
| *Groves, E. W. H., M.D., M.S. Lond. ... ... | 25 Victoria Square, Clifton. |

| | |
|---|---|
| *HAILES, CLEMENTS D. G., M.D., C.M. Ed. ... | 27 Alma Road, Clifton. |
| *HALL, E. G., M.B. Lond. ... ... ... ... ... | 42 Apsley Road, Clifton, Bristol. |
| *HALL, I. WALKER, M.D., Ch.B.Vict. ... ... | Linden Gate, Clifton Down Road, Clifton. |
| HANDFIELD-JONES, MONTAGU, M.D. Lond. ... | 36 Cavendish Square, London, W.1. |
| *HARRIS, H. ELWIN, B.A., M.B. Cantab. ... ... | 13 Lansdown Place, Clifton. |
| *HARSANT, W. H. ... ... ... ... ... ... ... | Tower House, Clifton Down Road, Clifton. |
| *HARTY, J. P. I., M.B., B.Ch., B.A.O., R.U.I. | 5 Westbourne Place, Clifton. |
| HAWES, C. S. ... ... .. ... ... ... ... ... | 8 Elsworthy Road, London, N.W.3. |
| HAYMAN, HOWARD ... ... ... ... .. ... ... | 3 Clifton Park, Clifton. |
| HELE, T. S., M.A., M.B. ... ... ... ... ... | Emmanuel College, Cambridge. |
| *HERAPATH, C. E. K., M.D. Lond. ... ... ... | 12 Brunswick Square, Bristol. |
| *HERAPATH, C. K. C. ... ... ... ... ... ... | Stoneleigh, Cotham Park, Bristol. |
| HILL, HEDLEY, M.D. Durh. ... ... ... ... | 1 Whiteladies Road, Clifton. |

HOSPITAL, BRISTOL GENERAL (*Secretary*, T. W. GREGG).

| | |
|---|---|
| *ILES, A. E., M.B., B.S. Lond. ... ... ... ... | Cotham Lawn, Cotham Park, Bristol. |
| *IMLAY, G. A., M.B., C.M. Aberd. ... ... ... | 4 Pembroke Vale, Clifton. |

INFIRMARY, BRISTOL ROYAL (*Secretary*, W. E. BUDGETT).

| | |
|---|---|
| INGLE, C. DURBAN ... ... ... ... ... ... ... | Somerton, Somerset. |

| | |
|---|---|
| *JAMES, C. W. W. ... ... ... ... ... ... ... | 246 Wells Road, Knowle, Bristol. |
| JAMISON, A., M.B., B.Ch., M.A. R.U I. ... ... | Ardenvohu Woodstock Road, Belfast. |
| *JOLL, C. A., M.B., M.S. Lond. ... ... ... ... | 21 Wimpole St., Cavendish Sq., W.1. |
| *JONES, J. ELLINGTON ... ... ... ... .. ... | 8 Chantry Road Clifton. |
| JOSEPH, A. HILL, M.D. Lond. ... ... ... ... | Bexhill-on-Sea. |

| | |
|---|---|
| *KAY-MOUAT, J. R., M.A. Oxon., M.B., Ch.B. Bristol ... ... ... ... ... ... ... ... ... ... | |
| *KERFOOT, STANLEY J. ... ... ... ... ... ... | 109 East Street, Bedminster, Bristol. |
| *KERR, R. A., M.B., B.Ch. ... ... ... ... ... | 40 Orients Gardens, Belfast, Ireland. |
| *KYLE, H. G., M.A., M.D. B.Ch. Oxon. ... ... | 31 Westbury Road, Bristol. |

| | |
|---|---|
| *LACE, F. ... ... ... ... ... ... ... ... ... | 5 Gay Street, Bath. |
| *LANSDOWN, R. G. P., M.D., B.S. Durh. ... ... | 39 Oakfield Road, Clifton. |
| *LAVINGTON, C. C., M.B., B.S. Durh. ... ... | 1 Burlington Road, Redland, Bristol. |
| LENDON, A. A., M.D. Lond. ... ... ... ... | North Terrace, Adelaide, Australia. |
| *LEES, E. LEONARD, M.D., C.M. Ed. ... ... ... | 1 Chesterfield Place, Clifton. |

### LIST OF SUBSCRIBERS.

| | |
|---|---|
| LEIGH, W. W. ... ... ... ... ... ... ... ... ... | Treharris, Glamorganshire. |
| *LEONARD, R. C., M.D. Durham ... ... ... ... | 4 Royal York Crescent, Clifton. |
| *LEWIS, D. J. ... ... ... ... ... ... ... ... ... | Glendower, Winscombe, Som. |
| *LINTON, MARION S., M.B. Lond. ... ... ... | 21 Oakfield Road, Clifton. |
| *LLEWELLYN, R. LL. J., M.B. Lond. ... ... ... | 41 Gay Street, Bath. |
| *LONGLEY, J. A. NOEL, M.B., Ch.B. Birm. ... | 124a Redland Road, Bristol. |
| *LUCAS, J. J. S., M.D. Lond. ... ... ... ... ... | 186 Wells Road, Totterdown, Bristol. |

| | |
|---|---|
| MACKINNON, CHARLES, M.B., C.M., M.A. Glasg. | Davaar, Cirencester. |
| MACLEAN, EWEN J., M.D., C.M. Ed.... ... ... | 12 Park Place, Cardiff. |
| MACLEAN, J. CAMPBELL, M.B., C.M. Ed.... ... | Swindon House, Swindon, Wiltshire. |
| *MACLEOD, DONALD, M.B., C.M. Ed. ... ... | 84 Redland Road, Redland, Bristol. |
| *MACWATTERS, J. C. .. ... ... ... ... ... | Almondsbury, near Bristol. |
| MARSHALL, ARTHUR L., M.B., B.A. Cantab. ... | Thornleigh, Upper Clapton, London, E.5. |
| *MARTIN, E. F., M.D., C.M. Ed. ... ... ... ... | 7 Royal Terrace, Weston-super-Mare. |
| *MARTYN, G. J. KING, M.D., B.C., B.A. Cantab. ... ... ... ... ... ... ... ... ... | 8 Gay Street, Bath. |
| MASON, S. B. ... ... ... ... ... ... ... ... ... | 12 Park Terrace, Pontypool. |
| MIALL, C. L'O. ... ... ... ... ... ... ... ... | Elm Hayes, Paulton, near Bristol. |
| *MOORE, C. A., M.B., M.S. Lond.... ... ... ... | 56 St. Paul's Road, Clifton. |
| MORRIS, L. N., M.B., Ch.B. Bris. ... ... ... | 24 All Hallows Road, Upper Easton, near Bristol. |
| *MORRIS, MARY E. H., M.D. Lond. ... ... ... | 19 Gay Street. Bath. |
| *MORTON, C. A., F.R.C.S. Eng. ... ... ... ... | 14 Vyvyan Terrace, Clifton. |
| MUNDEN, CHARLES ... ... ... ... ... ... ... | Ilminster, Somerset. |
| *MUNRO, J. M. H., D.Sc. Lond. ... ... ... ... | 12 Grosvenor Place, Bath. |
| *MYLES, G T. ... ... ... ... ... ... ... ... ... | 7 Apsley Road, Clifton. |

| | |
|---|---|
| *NEILD, NEWMAN, M.B., B.Ch. Vict. ... ... ... | 9 Richmond Hill, Clifton. |
| *NEWNHAM, W. H. C., M.B., M.A. Cantab. ... | 3 Lansdown Place, Victoria Square, Clifton. |
| *NICHOLS, F. C., M.B., Ch.B. Bristol ... ... ... | 38 Whiteladies Road, Clifton. |
| *NIXON, J. A., B.A., M.D., B.C. Cantab. ... ... | 7 Lansdown Place, Clifton. |
| *NIXON, J. H., M.D., B.S. Lond. ... ... ... | Morley House, Bath Road, Chippenham. |
| *NOEL-COX, H. L., M.D Durham ... ... ... | P. O. W. Military Hospital, Belmont, Surrey. |

| | |
|---|---|
| OPPENHEIMER, SON & Co. LTD. ... ... ... | 179 Queen Victoria Street, London, E.C.4. |
| *ORMEROD, H. LAWRENCE, M.B., B.Ch. R.U.I. | 2 Henleaze Road, Westbury Park, Bristol. |
| *ORR-EWING, H. J., M.B., B.S. Lond. ... ... | Halmer, South Road, Weston-snper-Mare. |

| | |
|---|---|
| PAGE, G. SHEPLEY ... ... ... ... ... ... ... ... | 78 Old Market Street, Bristol. |
| *PARKER, GEORGE, M.D., M.A. Cantab. ... ... | 14 Pembroke Road, Clifton. |
| PARKINSON, Capt. G. S., R.A.M.C. ... ... ... | |
| *PARSONS, H. F. ... ... ... ... ... ... ... ... | The Heath, Hazelwood Road, Sneyd Park. |

### LIST OF SUBSCRIBERS.

| | |
|---|---|
| Parsons, J. H., M.B., B.S., D.Sc. Lond.... ... | 54 Queen Anne Street, London, W. |
| Paterson, Donald Rose, M.D., C.M. Ed. ... | 15 St. Andrew's Crescent, Cardiff. |
| Phillips, C. M., M.D. Brux. ... ... ... ... | 502 Bath Road. |
| *Pickering, C. F. ... ... ... ... ... ... ... ... | 13 Berkeley Square, Bristol. |
| *Pineo, E. G. D.... ... ... ... ... ... ... ... ... | Richmond House, Langford, nr. Bristol |
| Powell, J. J., M.D. Lond. ... ... ... ... ... | 2 Gloucester Road, Bishopston, Bristol. |
| Price, D. T. M.B. Lond. ... ... ... ... ... | Castle Cary, Somerset. |
| *Prichard, Arthur W. ... ... ... ... ... ... | 6 Rodney Place, Clifton. |
| *Prowse Arthur B., M.D. Lond. ... ... ... | 5 Lansdown Place, Clifton. |

| | |
|---|---|
| *Rattray, J. M., M.D., C.M., M.A. Aberd. ... | Rook Lane House, Frome, Somerset. |
| *Rayner, D. C., F.R.C.S. Eng. ... ... ... ... | 9 Lansdown Place, Clifton, Bristol. |
| *Richardson, T. A. ... ... ... ... ... ... ... | 10 Clifton Park Road, Clifton. |
| Ritchie, Thomas P. ... ... ... ... ... ... | 27 Oakfield Road, Clifton. |
| *Robertson, D., M.B., Ch.B. Edin. ... ... ... | 205 Wells Road, Knowle. |
| *Rogers, B. M. H., M.D., B.Ch., B.A. Oxon. ... | 14 Mortimer Road, Clifton. |
| *Rolfe, R., B.A., M.B., B.C. Cantab. ... ... | Park House, St. Andrews Road, Avonmouth. |
| *Rose, F. H. ... ... ... ... ... ... ... ... ... | Westfield, Portishead, Somerset. |
| *Rossiter, George F., M.B. Lond. ... ... ... | Cairo Lodge, Weston-super-Mare. |
| *Rudge, C. King ... ... ... ... ... ... ... .. | 145 Whiteladies Road, Bristol. |
| *Rutherford, J. M., M.B., C.M. Edin., F.R.C.P. | Brislington House, near Bristol. |

| | |
|---|---|
| Seward, W. J., M.B. Lond. ... ... ... ... | 15 Chandos Avenue, Oakleigh Park, London, N. |
| *Short, A. R., M.D., B.S., B.Sc. Lond. ... ... | Montrose House, Queen's Road, Clifton |
| *Short, L. J., M.D., B.S. Lond., M.D. Bris. ... | Eton Villa, The Shrubbery, Weston-super-Mare. |
| *Smith, G. Cockburn, M.D. Brux. ... ... ... | 14 South Road, Newton Abbot. |
| Smith, L. Shingleton, B.A., M.B. Cantab.... | Camden Road, Brecon. |
| Smith, Rev. Reginald, M.A. Cantab. ... ... | The Vicarage, Walpole St. Andrew Norfolk. |
| Smith, W. Steele, M.B. Lond. ... ... ... | Caldecote, Cambridge. |
| *Smith, W. Alexander, M.B., M.A. Oxon. ... | 70 Pembroke Road, Clifton. |
| Society, Cardiff Medical (*Hon. Lib.*, Queen Street, Cardiff). | |
| Society, Manchester Medical ... ... ... | Medical Society's Library, The Owens College, Manchester. |
| *Stack E. H. E., B.A., M.B., B.C. Cantab. ... | Arvalee, Clifton Down Road, Clifton. |
| Staple, J. D. ... ... ... ... ... ... ... ... ... | St Martin's, Lyme Regis, Dorset. |
| *Statham, R. S. S., M.D. Bristol ... ... ... | 5 Westbourne Place, Clifton. |
| *Stock, W. S. V., M.B., B.S. Lond. ... ... ... | 1 Mortimer Road, Clifton. |
| *Swain, James, M.D., M.S. Lond., C.B. ... ... | 4 Victoria Square, Clifton. |
| *Swayne, W. Carless, M.D. Lond. ... ... ... | 2 Clifton Park, Clifton. |
| *Symes, J. O., M.D. Lond. ... ... ... ... ... | 71 Pembroke Road, Clifton. |
| Symonds, H. P. ... ... ... ... ... ... ... ... | 35 Banbury Road, Oxford. |

### LIST OF SUBSCRIBERS.

| | |
|---|---|
| TAYLOR, A. L. ... ... ... .. ... ... ... ... | The Royal Infirmary, Bristol. |
| TAYLOR, H. N., M.A. Cantab., M.D. Dublin ... | Hillside, Ebbw Vale. |
| *TAYLOR, JAMES ... ... ... ... ... ... ... ... | 36 Alma Road, Clifton |
| *TAYLOR, J. W., M.D. Bristol ... ... ... ... | 8 Redcliff Parade West, Bristol. |
| *TAYLOR, S. H. S., B.A. Cantab., M.B., Ch.B.Ed. | Wray Croft, Lacock, Chippenham. |
| *TEMPLE, G. H., M.B., C.M. Ed. ... ... ... | Ailanthus, Bristol Road, Weston-super-Mare. |
| THIN, JAMES ... ... ... ... ... ... ... ... ... | 54 & 55 South Bridge, Edinburgh. |
| *THOMAS, J. D., M.B. Cantab. ... ... ... ... | Northwoods, Winterbourne. |
| THOMAS, J. LYNN, C.B. ... ... ... ... ... ... | Green Lawn, Pen-y-lan, Cardiff. |
| *THOMAS, J. TUBB, D.P.H. ... ... ... ... ... | The Halve House, Trowbridge. |
| *THOMAS, R. E., M.D. B.S. Lond. ... ... ... | Hospital and Sanatorium, Ham Green, Pill. |
| *THOMSON, F. G., M.A., M.D. Lond. ... ... | 20 Gay Street, Bath. |
| TOSSWILL, LEONARD R., D.P.H. Oxon. ... | |
| VACHELL, HERBERT R., M.D., C.M. Aberd. ... | 18 Newport Road, Cardiff. |
| *VICKERY, W. H. ... ... ... ... ... ... ... ... | 1 Trewartha Park, Weston-super-Mare |
| *WALDO, HENRY, M.D., C.M. Aberd. ... ... ... | 19 Pembroke Road, Clifton. |
| *WALKER, CYRIL H., M.B., B.A. Cantab. ... ... | 8 Oakfield Road, Clifton. |
| *WALLACE, JOHN ... ... ... ... ... ... ... ... | Lauriston, 56 Knightstone Road, Weston-super-Mare. |
| WALLACE, J. T., M.B., B.Ch., B.A. R.U.I. ... | 48 Coronation Road, Bedminster, Bristol. |
| *WALTERS, C. F. ... ... ... ... ... ... ... ... | 5 Mortimer Road, Clifton. |
| *WATERHOUSE, R., M.D. Lond. .. ... .. ... | 25 Circus, Bath. |
| *WATSON-WILLIAMS, P., M.D. Lond.... ... ... | 2 Rodney Place, Clifton. |
| *WHICHER, A. H. ... ... ... ... ... ... ... ... | 115 Queen's Road, Clifton. |
| WHITE, J. W. ... ... ... ... ... ... ... ... ... | Orchard House, Nailsea. |
| *WHITE, PERCY W. ... ... ... ... ... ... ... ... | 37 Whiteladies Road, Clifton. |
| WIGAN, C. L., M.B., B.S. Durham ... ... ... | Clarence House, Portishead. |
| WIGMORE, J., M.D. R. U. I.... ... ... ... ... | 16 Queen Square, Bath. |
| WILLCOX, R. LEWIS ... ... ... ... ... ... ... | 97 London Road, Salisbury, Wilts. |
| WILLETT, GEORGE G. D. ... ... ... ... ... ... | Keynsham, Somerset. |
| *WILLIAMS, E. C., M.B., B.C., B.A. Cantab. ... | 159 Whiteladies Road, Clifton. |
| WILLIAMS, H. EGERTON ... ... ... ... ... ... | The Mount, Newport, Mon. |
| WILLIAMS, S. R., M.B. Lond. ... ... ... ... | Buckfastleigh, Devon. |
| *WILLIAMSON, G. SCOTT... ... ... ... ... ... | The General Hospital, Bristol. |
| WILLIS, W. M.... ... ... ... ... ... ... ... ... | 7 Regent Street, Nottingham. |
| *WILLS, W. K., M.B., B.C. Cantab. ... ... ... | 19 Whiteladies Road, Clifton. |
| *WINDSOR-AUBREY, H. W. ... ... ... ... ... | 79 Coronation Road, Bristol. |
| *WOOD, DUNCAN ... ... ... ... ... ... ... ... | The Porch, Corsham, Wilts. |
| *WOOD, W. V. ... ... ... ... ... ... ... ... ... | Langford, near Bristol. |
| *WRIGHT, A. J., M.B., B.S. Lond. ... ... ... | 14 Victoria Square, Clifton. |
| YOUNG, J., M.D. Glas. ... ... ... ... ... ... ... | |

